# Capillasterin A, a Novel Pyrano[2,3-f]chromene from the Australian Crinoid *Capillaster multiradiatus*

**DOI:** 10.3390/md17010026

**Published:** 2019-01-04

**Authors:** Kah Yean Lum, Anthony R. Carroll, Merrick G. Ekins, Silven Read, Zahra Haq, Ian Tietjen, James St John, Rohan A. Davis

**Affiliations:** 1 Griffith Institute for Drug Discovery, Griffith University, Brisbane, QLD 4111, Australia; kahyean.lum@griffithuni.edu.au (K.Y.L.); a.carroll@griffith.edu.au (A.R.C.); j.stjohn@griffith.edu.au (J.S.J.); 2 Biodiversity and Geosciences, Queensland Museum, South Brisbane BC, QLD 4101, Australia; merrick.ekins@qm.qld.gov.au; 3 Faculty of Health Sciences, Simon Fraser University, Burnaby, BC V5A 1S6, Canada; silven_read@sfu.ca (S.R.); zahra_haq@sfu.ca (Z.H.); ian_tietjen@sfu.ca (I.T.)

**Keywords:** Crinoid, feather star, *Capillaster multiradiatus*, naphthopyrones, pyrano[2,3-f]chromene, capillasterin A, HIV-1

## Abstract

Capillasterin A (**1**), a novel pyrano[2,3-f]chromene, together with seven known naphthopyrones including comaparvin (**2**), TMC-256C1 (**3**), 6-methoxycomaparvin-5- methyl ether (**4**), 5,8-dihydroxy-6-methoxy-2-propyl-4H-naphtho[2,3-b]pyran-4-one (**5**), 5,8-dihydroxy-6,10-dimethoxy-2-propyl-4H-naphtho[2,3-b]pyran-4-one (**6**), TMC-256A1 (**7**) and 6-methoxycomaparvin (**8**) were isolated from an EtOH/H_2_O extract from the Australian crinoid *Capillaster multiradiatus*. The structures of all the compounds were determined by detailed spectroscopic (1D/2D NMR and MS) data analysis. This is the first report of a natural product that contains the pyrano[2,3-f]chromene skeleton. Compounds **2**–**6** were observed to display moderate inhibition of in vitro HIV-1 replication in a T cell line with EC_50_ values ranging from 7.5 to 25.5 µM without concomitant cytotoxicity.

## 1. Introduction

Crinoids belong to the Crinoidea class and are the most primitive group of present-day echinoderms [1]. To date, approximately 700 crinoid species have been identified, and they typically exist in two forms, which include the unstalked feather stars and the stalked sea lilies [2]. Due to our continuing interest in crinoid-derived natural product chemistry [3], the feather star *Capillaster multiradiatus* (Linnaeus, 1758), which belongs to the family Comatulidae, was selected for chemical investigations, since no studies had been undertaken on this species collected from Australian waters. Comatulidae can be easily distinguished from other feather stars by two apomorphic features, such as the proximal pinnule combs and a marginal mouth in the adult [4]. Crinoids of the family Comatulidae, for instance *Comanthus* sp., have been shown to produce secondary metabolites such as anthraquinones and naphthopyrones, which possess various biological activities, including cytotoxicity against tumour cell lines [5], inhibition of NF-κB activation that is responsible for cancer development and inflammation [6], as well as anti-inflammatory and analgesic activities [7]. Naphthopyrones TMC-256A1 and 5,8-dihydroxy-6,10-dimethoxy-2-propyl-4H-naphtho[2,3-b]pyran-4-one have previously been isolated from *Capillaster multiradiatus*, and these compounds were found to inhibit the multidrug transporter ABCG2 that is responsible for drug resistance [8]. In addition, Jebasingh et al. [9] reported that the extracts of *C. multiradiatus* collected from the Tuticorin Coast, India exhibited antibacterial activity against several fish and human pathogenic bacterial strains. A recent report showed that a Vietnamese *C. multiradiatus* also produces anthraquinones, as well as butenolides [10]. In this study, we report the isolation of seven known naphthopyrones and a novel pyrano[2,3-f]chromene natural product from an Australian collection of *C. multiradiatus*, as well as some biological activities for the isolated compounds. 

## 2. Results and Discussion

**Figure 1:**
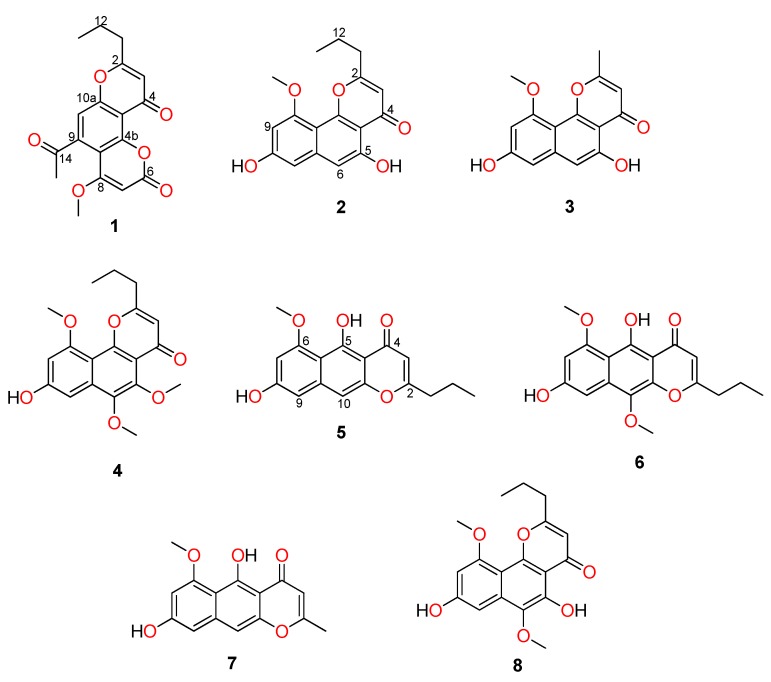
Chemical structures of capillasterin A (**1**) and naphthopyrones (**2–8**) isolated from the Australian crinoid *C. multiradiatus.*

The 70:30 EtOH/H_2_O extract of *C. multiradiatus* was subjected to diol-bonded silica flash column chromatography using a stepwise solvent gradient from 100% *n*-hexane to 100% EtOAc, followed by 100% MeOH. The fractions containing UV-active compounds were purified using normal-phase HPLC (NP-HPLC) or reversed-phase HPLC (RP-HPLC). For NP-HPLC, diol semi-preparative HPLC was utilised with a solvent system of i-PrOH/*n*-hexane whereas for RP-HPLC, a solvent system of H_2_O/MeOH/0.1%TFA was used. Based on the LC-MS and NMR spectroscopic data, along with comparisons to literature values, seven known naphthopyrones, including comaparvin (**2**) [11], TMC-256C1 (**3**) [12], 6-methoxycomaparvin-5-methyl ether (**4**) [6], 5,8-dihydroxy-6-methoxy-2-propyl-4H-naphtho[2,3-b]pyran-4-one (**5**) [13], 5,8-dihydroxy-6,10-dimethoxy-2-propyl-4H-naphtho[2,3-b]pyran-4-one (**6**) [14], TMC-256A1 (**7**) [12], and 6-methoxycomaparvin (**8**) [6], were identified, along with one novel compound that was given the trivial name capillasterin A (**1**)(Figure 1).

Capillasterin A (**1**) was isolated as a yellow amorphous powder and was assigned the molecular formula C_18_H_16_O_6_ following analysis of the (+)-HRESIMS ion at *m/z* 351.0839 [M + Na]^+^ (calcd. for C_18_H_16_O_6_Na, 351.0839). The ^1^H NMR spectrum (Figure S1) of capillasterin A in CDCl_3_ revealed signals corresponding to three aromatic protons (δ_H_ 7.06, 6.21, and 5.76), three methyl groups (δ_H_ 3.96, 2.52, 1.03), and two methylene protons (δ_H_ 2.58, 1.76) (Table 1). The ^13^C NMR and edited HSQC spectra (Figures S2 and S4) suggested the presence of 18 carbon atoms, including three methyls, two methylenes, three methines, and ten non-protonated sp^2^ carbons. The ^13^C NMR resonances at δ_C_ 175.2, 161.0 and 201.2 indicated that capillasterin A most probably contained three carbonyl functional groups, and this was supported by the IR data, which displayed absorbances between 1731 to 1659 cm^−1^. 

**Figure 2:**
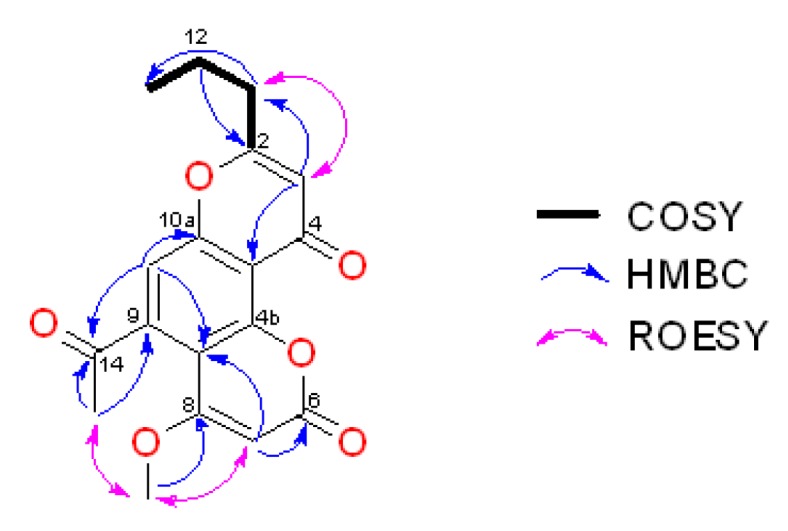
COSY and key HMBC/ROESY correlations for capillasterin A (**1**).

**Figure 3:**
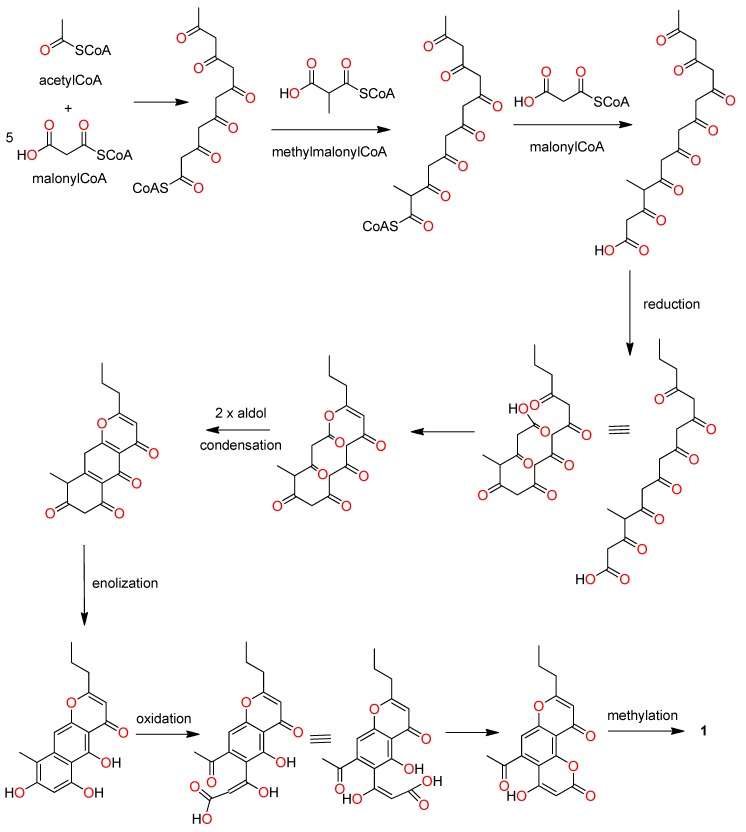
Proposed biosynthetic pathway for capillasterin A (**1**).

The ^1^H–^1^H COSY displayed one spin system that included an *n-*propyl chain (–CH_2_CH_2_CH_3_) attached to a trisubstituted double bond (Table 1, Figure 2 and Figure S3). This system was further expanded to a γ-pyrone after analysis of HMBC data, specifically, HMBC correlations from δ_H_ 6.21 to δ_C_ 35.6, 113.1, 168.0, and 175.2 (Table 1, Figure 2 and Figure S5). This *n-*propyl substituted γ-pyrone was supported by comparison of NMR data with that reported for the known crinoid natural products comaparvin (**2**) [11] and 6-methoxycomaparvin (**8**) [6]. The HMBC correlations observed from δ_H_ 7.06 to δ_C_ 107.9, 113.1, 159.2, 153.2 and 201.2 suggested **1** contained an acylated aromatic group. The presence of an acetophenone was supported by the HMBC correlations from the methyl protons (δ_H_ 2.52) to a carbonyl carbon at δ_C_ 201.2 and to an aromatic carbon at δ_C_ 143.8. The HMBC correlations of δ_H_ 6.21 and δ_H_ 7.06 to δ_C_ 113.1, and δ_H_ 7.06 to δ_C_ 175.2 suggested that the *n*-propyl substituted γ-pyrone was linked to the acetophenone at C-4a and C-10a. In comparison with the ^13^C chemical shift of the aromatic carbons at C-3 and C-10, C-7 had a distinct upfield chemical shift at δ_C_ 90.6, suggesting it was vicinal to an oxygenated carbon. This observation was consistent with the HMBC correlations of the methoxy protons (δ_H_ 3.96) to δ_C_ 164.9 and δ_C_ 90.6 (weak). Analysis of ROESY data further supported this assignment (Table 1, Figure 2 and Figure S6). The proton resonating at δ_H_ 5.76 (H-7) also showed a weak HMBC correlation to δ_C_ 161.0, indicating the presence of a carbonyl moiety at C-6. Based on the calculated double bond equivalents (DBE = 11) and the molecular formula assigned by (+)-HRESIMS, an additional oxygen linkage between C-4b and C-6, forming an α,β-unsaturated lactone was proposed. The ^13^C chemical shifts of C-4b (δ_C_ 153.2) and C-6 (δ_C_ 161.0) are consistent with that expected for related carbons in a coumarin [11]. With this final linkage in place, the full structure of **1**, 5-acetyl-4-methoxy-8-propylpyrano[2,3-f]chromene-2,10-dione, was assigned. Capillasterin A, along with the other compounds reported in this crinoid study, are all polyketide-derived metabolites [15,16]; a plausible biosynthetic route from acetyl CoA and malonyl CoA is shown below in Figure 3.

The ability of compounds **1**–**7** to inhibit HIV-1 replication was evaluated in vitro using an established multi-cycle replication assay [17]. Briefly, the CEM-GXR T cell line, which contains an HIV-1 promoter-driven green fluorescent protein (GFP) reporter, was infected with the full-length, infectious HIV-1 strain NL4.3 at a multiplicity of infection of 0.03 [18]. After 24 hours, cells were washed and subsequently incubated with compounds **1**–**7**, the control HIV inhibitor efavirenz, or 0.1% DMSO vehicle control. GFP expression, a marker of viral replication, was then measured by flow cytometry after 72 hours. In this assay, compound **2** was the most active with an EC_50_ of 7.5 ± 1.7 µM, while compounds **3**–**6** exhibited EC_50_ ranging from 14.5 ± 5.8 to 25.5 ± 3.4 µM (Table 2). Consistent with our previous results [17], the positive control efavirenz inhibited HIV-1 replication with an EC_50_ of 0.0025 ± 0.0019 µM. 6-Methoxycomaparvin (**8**) was not tested since this molecule proved to be unstable.

Compounds **1**–**7** were further assessed for cellular toxicity in CEM cells, the parental cell line of CEM-GXR. After 72 hours’ treatment with compounds, toxicity was assessed by measuring the surface expression of the early apoptotic marker annexin V by flow cytometry (by staining with annexin V-APC). In this assay, treatment of cells with 3 µM of the control histone deacetylase inhibitor panobinostat resulted in a 4.2 ± 1.8-fold increase in apoptotic cells relative to cells treated with 0.1% DMSO, consistent with previous results [19]. In contrast, no increase in apoptosis was observed up to 100 µM for compounds **1**–**7**, with the exception of compound **2**, which induced 1.4 ± 0.2 and 2.2 ± 0.4-fold increases at 30 and 100 µM, respectively.

In regard to the structure activity relationship (SAR), our initial screening of naphthopyrones on this assay shows that the angular forms of naphthopyrones exhibited improved inhibition against HIV-1 replication than the linear forms. For instance, compound **2** demonstrated an approximately 2.0-fold increase in activity as compared to **5**. Similarly, compound **3** showed weaker but consistent antiviral activity, while **7** exhibited no detectable activity (Table 2). Furthermore, we noticed that compound **3**, which only differs from **2** by the substitution of a methyl instead of a propyl group at C-2, showed a 3.4-fold reduction in activity. A similar trend was also observed for the linear naphthopyrones **5** and **7**, where **7** had loss of activity due to a minor substitution on C-2. Therefore, based on our preliminary SAR studies, together with SAR of naphthopyrones on the inhibition of ABCG-2-mediated transport reported by Bokesch et al. [8], we also postulate that the propyl group in naphthopyrones plays an important role for biological activities.

The three most abundant compounds, capillasterin A (**1**), 6-methoxycomaparvin-5-methyl ether (**4)** and TMC-256A1 (**7**) were also tested for their ability to stimulate the proliferation of GFP-expressing immortalised mouse olfactory ensheathing cells (mOEC) using a cell proliferation assay; none of the compounds showed a significant increase in mOEC viability at 10 µM after 24 hours of treatment. Compounds **1**–**7** have now been added to the Davis Open-Access Compound Library housed at Compounds Australia [20], Griffith University, where they are available for collaboration in drug discovery [21] or chemical biology research [22].

## 3. Materials and Methods

### 3.1. General Chemistry Experimental Procedures

NMR spectra were recorded at 25 °C on a Bruker AVANCE III HD 800 MHz NMR spectrometer (Zürich, Switzerland) equipped with a cryoprobe. The ^1^H and ^13^C chemical shifts were referenced to the solvent peaks for CDCl_3_ at δ_H_ 7.26 and δc 77.16, respectively. LRESIMS data were recorded on a Waters ZQ ESI mass spectrometer (Milford, MA, USA). HRESIMS data were acquired on a Bruker maXis II ETD ESI-qTOF (Bremen, Germany) and the mass spectrum was calibrated externally with 0.1 mg/mL of sodium trifluoroacetate. UV spectra were recorded using a JASCO V-650 UV/vis spectrophotometer (Easton, MD, USA). IR data were acquired on Universal Attenuated Total Reflectance (UATR) Two attachment on a PerkinElmer spectrometer (Waltham, MA, USA). Alltech Davisil 30–40 µm 60 Å diol-bonded silica (NSW, Australia) was packed into an open glass column (9 × 3.8 cm) for diol flash column chromatography. Preparative thin layer chromatography (PTLC) was carried out on Merck silica gel 60 F_254_ pre-coated aluminium plates (Darmstadt, Germany) and was observed using UV light (254 nm). Alltech C_18_ bonded silica (35–75 µm, 150 Å) (NSW, Australia) was used for pre-adsorption work before HPLC separations, and the pre-absorbed sample was packed into an Alltech stainless steel guard cartridge (10 × 30 mm) (NSW, Australia). A Waters 600 pump fitted with a Waters 996 photodiode array detector (Milford, MA, USA) and Gilson 717-plus autosampler (Middleton, WI, USA) was used for semi-preparative HPLC separations. Thermo Electron Betasil C_18_ (150 × 21.2 mm) (Waltham, MA, USA) and YMC-Pack Diol-120-NP (150 x 20 mm) (Kyoto, Japan) columns were used for semi-preparative HPLC separations. All solvents used for chromatography, UV and MS were Honeywell Burdick & Jackson HPLC grade (Muskegon, MI, USA). H_2_O was filtered using Sartorius Arium® Pro VF ultrapure water system (Göttingen, Germany).

### 3.2. Collection, Extraction and Isolation

*C. multiradiatus* was collected on the 28 January 2004 by trawling at a depth of 37 m in the Torres Strait (9°31′12″ South 143°45′36″ East), Queensland, Australia by CSIRO on the ship RV *Gwendoline May*, which was part of the SBD project. The voucher specimen (QM G236364) was deposited at the Queensland Museum, South Brisbane, Queensland Australia. The sample was immediately frozen at a temperature of −20 °C upon collection and then stored in 70% EtOH/30% H_2_O.

The EtOH/H_2_O (7:3) extract of *C. multiradiatus* obtained was dried under rotary evaporation to give a red extract (287.9 mg). The extract was subjected to diol-bonded silica flash column chromatography using a stepwise solvent gradient from 100% *n*-hexane to 100% EtOAc, followed by 100% MeOH. Twelve fractions (12 × 100 mL) were collected and analysed by ^1^H NMR spectroscopy. Fraction 6 (10.1 mg) eluted in 50% EtOAC-50% *n*-hexane was pre-absorbed onto diol-bonded silica gel, packed into a guard cartridge, and this column was then attached to a semi-preparative diol HPLC column (150 × 20 mm). A linear gradient from 100% *n*-hexane to 20% i-PrOH–80% *n*-hexane was performed over 60 min. Sixty fractions (60 × 1 min) were collected from the start of the HPLC run. 6-methoxycomaparvin (**8**, 0.3 mg *t*_R_ 44 min), comaparvin (**2**, 1.3 mg, *t*_R_ 46–47 min), TMC-256C1 (**3**, 0.1 mg, *t*_R_ 54 min) and TMC-256A1 (**7**, 1.3 mg, *t*_R_ 56–57 min) were isolated. The fraction eluting at *t*_R_ 49 min (2.5 mg) was re-purified using preparative thin layer chromatography with 30% EtOAC-70% *n*-hexane, affording 5,8-dihydroxy-6-methoxy-2-propyl-4H-naphtho[2,3-b]pyran-4-one (**5**, 1.4 mg) and 5,8-dihydroxy-6,10-dimethoxy-2-propyl-4H-naphtho[2,3-b]pyran-4-one (**6**, 1.1 mg). Fraction 8 (6.7 mg) that eluted in 70% EtOAC-30% *n*-hexane was pre-adsorbed onto C_18_-bonded silica gel, packed into a guard cartridge, and then attached to a semi preparative Betasil C_18_ HPLC column. A linear gradient from 10% MeOH (0.1% TFA)–90% H_2_O (0.1% TFA) to 100% MeOH (0.1% TFA) at flow rate of 9 mL/min was performed over 60 min. 6-methoxycomaparvin 5-methyl ether (**4**, 3.4 mg, *t*_R_ 50–51 min) and the novel compound, capillasterin A (**1**, 2.0 mg, *t*_R_ 42–43 min) were obtained.

Capillasterin A (**1**): Yellow amorphous powder; UV (MeOH) λ_max_ (log ε) 202 (3.53), 253 (3.50), 326 (3.06) nm; IR (UTAR) λ_max_ 1731, 1708, 1659, 1628, 1590, 1477, 1459, 1431, 1374, 1308, 1261, 1160, 1109, 994, 966, 817 cm^−1^; ^1^H and ^13^C NMR data, Table 1; (+)-LRESIMS *m/z* 351 (100) [M + Na]^+^; (+)-HRESIMS *m/z* 351.0839 (100) [M + Na]^+^ (calcd. C_18_H_16_O_6_Na, 351.0839). 

### 3.3. HIV-1 Replication and Apoptosis Assays

The following reagents were obtained from the NIH AIDS Reagent Program: CEM CD4^+^ cells (CEM) from Dr. J.P. Jacobs, [23] and pNL4-3 from M. Martin [24]. Efavirenz and panobinostat were purchased from Sigma-Aldrich (St. Louis, MO, USA). CEM and CEM-derived GXR25 GFP-reporter T cells (CEM-GXR; [18]) were cultured in R10+ medium [RPMI-1640 with HEPES and l-Glutamine (Lonza, Basel, Switzerland), 10% fetal calf serum, 100 U of penicillin/mL, and 100 µg of streptomycin/mL Sigma-Aldrich (St. Louis, MO, USA)]. Production of HIV-1 NL4.3 and multi-cycle HIV-1 replication assays were performed as described previously [25]. Apoptosis assays were performed by staining CEM cells with annexin V-APC (BioLegend, San Diego, CA) according to manufacturer instructions. Results are presented as the mean ± s.d. from at least 3 independent experiments, with the exception of the apoptosis assays if no apoptosis was observed at 100 µM, which are from 2 independent experiments.

### 3.4. Cell Proliferation Assay

The cell proliferation assay was performed according to the method described by Chen et al. [26], with modification. Briefly, GFP-expressing immortalised mouse OECs (mOEC) were seeded at 2000 cells/well in a 384-well microplate (Greiner Bio-One, Kremsmünster, Austria) with a Multidrop™ Combi Reagent Dispenser (Thermo Fisher Scientific, Waltham, MA, USA) using the BioCel Automation System (Agilent Technologies, Santa Clara, CA, USA) and incubated overnight at 37 °C under 5% CO_2_. After 24 hours, the cells were treated with 4 different concentrations of pure compounds (10, 1, 0.1 and 0.01 µM). 0.2% DMSO was used as negative control. Plates were incubated for 24 hours at 37 °C under 5% CO_2._ Cell viability of mOECs was measured by adding 5 µL of resazurin (500 μM, Sigma-Aldrich, Sydney, Australia) to the cells and incubated in dark for 4 hours at 37 °C under 5% CO_2._ The dehydrogenase enzymes found in metabolically active cells will convert resazurin to resofurin products that are soluble in culture medium. The fluorescent resofurin products produced by the viable cells are quantified using an EnVision™ Multilabel (PerkinElmer, Waltham, MA, USA) plate reader at 535/595nm. After that, the cells were fixed and stained with Hoechst (Thermo Fisher Scientific, Waltham, MA, USA) and stored at 4 °C prior to imaging. Cells were imaged automatically using Operetta™ (PerkinElmer, Waltham, MA, USA). Individual cell segmentation and cell count were performed using the Harmony 3.5.2® software.

## 4. Conclusions

Chemical investigations of an Australian feather star *C. multiradiatus* extract led to the isolation and identification of seven known napthopyrones and a novel pyrano[2,3-f]chromene, capillasterin A (**1**). This is the first report of a natural product that contains the pyrano[2,3-f] chromene skeleton. Of the seven compounds tested, five naphthopyrones (**2**–**6**) were found to exhibit moderate inhibition on in vitro HIV-1 replication in a T cell line. 

## Figures and Tables

**Table 1:** NMR data for capillasterin A (**1**) in CDCl_3._*^a.^*

Position	δc, type	δ_H_, mult. (*J* in Hz)	COSY	HMBC	ROESY
2	168.0, C	-	-	-	-
3	112.7, CH	6.21, s	11	2, 4, 4a, 4b ^w^, 11	11
4	175.2, C	-	-	-	-
4a	113.1, C	-	-	-	-
4b	153.2, C	-	-	-	-
6	161.0, C	-	-	-	-
7	90.6, CH	5.76, s	8-OCH_3_^w^	6, 8, 8a, 9	8-OCH_3_
8	164.9, C	-	-	-	-
8a	107.9, C	-	-	-	-
9	143.8, C	-	-	-	-
10	111.2, CH	7.06, s	-	4 ^w^, 4a, 4b ^w^, 8a, 10a, 14	-
10a	159.2, C	-	-	-	-
11	35.6, CH_2_	2.58, t (7.2)	12	13, 12, 3, 2	3, 13
12	20.1, CH_2_	1.76, tq (7.2, 7.4)	11,13	13, 11, 2	-
13	13.6, CH_3_	1.03, t (7.4)	12	11, 12	11
14	201.2, C	-	-	-	-
15	31.4, CH_3_	2.52, s	-	9, 14	8-OCH_3_^w^
8-OCH_3_	56.7, OCH_3_	3.96, s	7 ^w^	8, 7 ^w^	7, 15 ^w^

*^a^* Spectra recorded at 25 °C and 800 MHz for ^1^H and 200 MHz for ^13^C; ^w^ = weak correlation.

**Table 2:** Activities of compounds **1**–**7** on in vitro HIV-1 replication in CEM-GXR T cells.

Compound	EC_50_ (µM) ^a^
**1**	>100
**2**	7.5 ± 1.7
**3**	25.5 ± 3.4
**4**	14.5 ± 5.8
**5**	15.6 ± 6.5
**6**	20.2 ± 4.9
**7**	>100
**Efavirenz**	0.0025 ± 0.0019

**^a^** Results are presented as mean ± s.d. of three independent experiments.

## Supplementary Materials

The following are available online at http://www.mdpi.com/1660-3397/17/1/26/s1, Figures S1–S6: ^1^H NMR, ^13^C NMR, COSY, HSQC, HMBC, ROESY spectra of capillasterin A (**1**).
