# Supplementary material for: Capillasterin A, a Novel Pyrano[2,3-f]chromene from the Australian Crinoid Capillaster multiradiatus
Source: Mar Drugs. 2019 Jan 4;17(1):26. doi: 10.3390/md17010026 (PMC6356231; doi:10.3390/md17010026)

Supplementary Materials Contents Page

Capillasterin A, a Novel Pyrano[2,3-f]chromene from the Australian Crinoid *Capillaster multiradiatus*

Kah Yean Lum [1], Anthony R. Carroll [1], Merrick G. Ekins [2], Silven Read [3], Zahra Haq [3], Ian Tietjen [3], James St John [1] and Rohan A. Davis [1,*]

[1] Griffith Institute for Drug Discovery, Griffith University, Brisbane, QLD 4111, Australia; kahyean.lum@griffithuni.edu.au (K.Y.L.); a.carroll@griffith.edu.au (A.R.C.); j.stjohn@griffith.edu.au (J.S.J.)

[2] Biodiversity and Geosciences, Queensland Museum, South Brisbane BC, QLD 4101, Australia; merrick.ekins@qm.qld.gov.au

[3] Faculty of Health Sciences, Simon Fraser University, Burnaby, BC, Canada; silven_read@sfu.ca (S.R.); zahra_haq@sfu.ca (Z.H.); ian_tietjen@sfu.ca (I.T);

* Correspondence: r.davis@griffith.edu.au; Tel: +61-7-3735-604; Fax: +61-7-3735-6001.

Contents:

Figure S1 ^{1}H NMR (800 MHz) spectrum of capillasterin A (**1**) in $CDCl_3$
Figure S2 ^{13}C NMR (200 MHz) spectrum of capillasterin A (**1**) in $CDCl_3$
Figure S3 COSY NMR spectrum of capillasterin A (**1**) in $CDCl_3$
Figure S4 HSQC NMR spectrum of capillasterin A (**1**) in $CDCl_3$
Figure S5 HMBC NMR spectrum of capillasterin A (**1**) in $CDCl_3$
Figure S6 ROESY NMR spectrum of capillasterin A (**1**) in $CDCl_3$

Figure S1 ^{1}H NMR (800 MHz) spectrum of capillasterin A (**1**) in $CDCl_3$

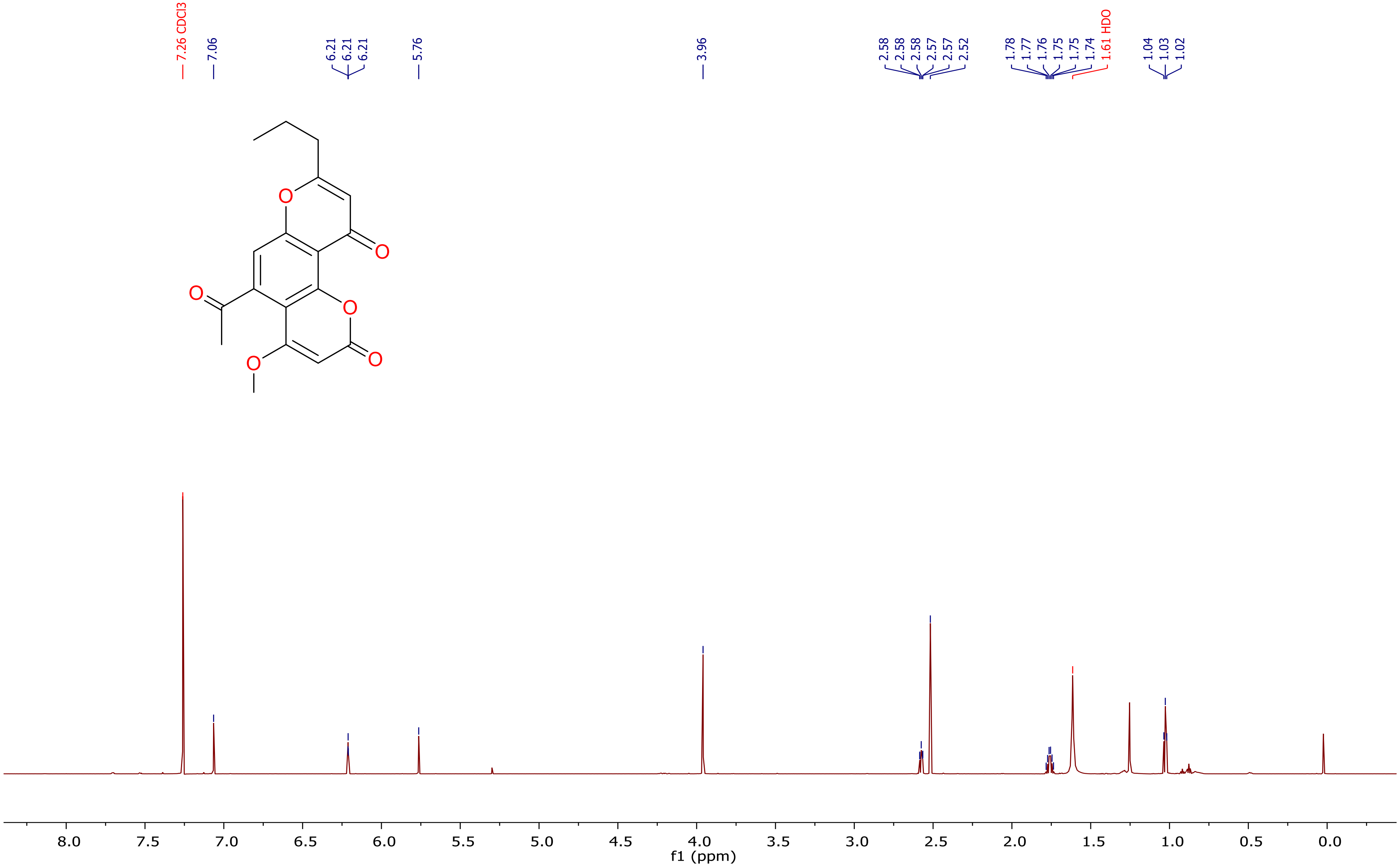

Figure S2 ^{13}C NMR (200 MHz) spectrum of capillasterin A (**1**) in $CDCl_3$

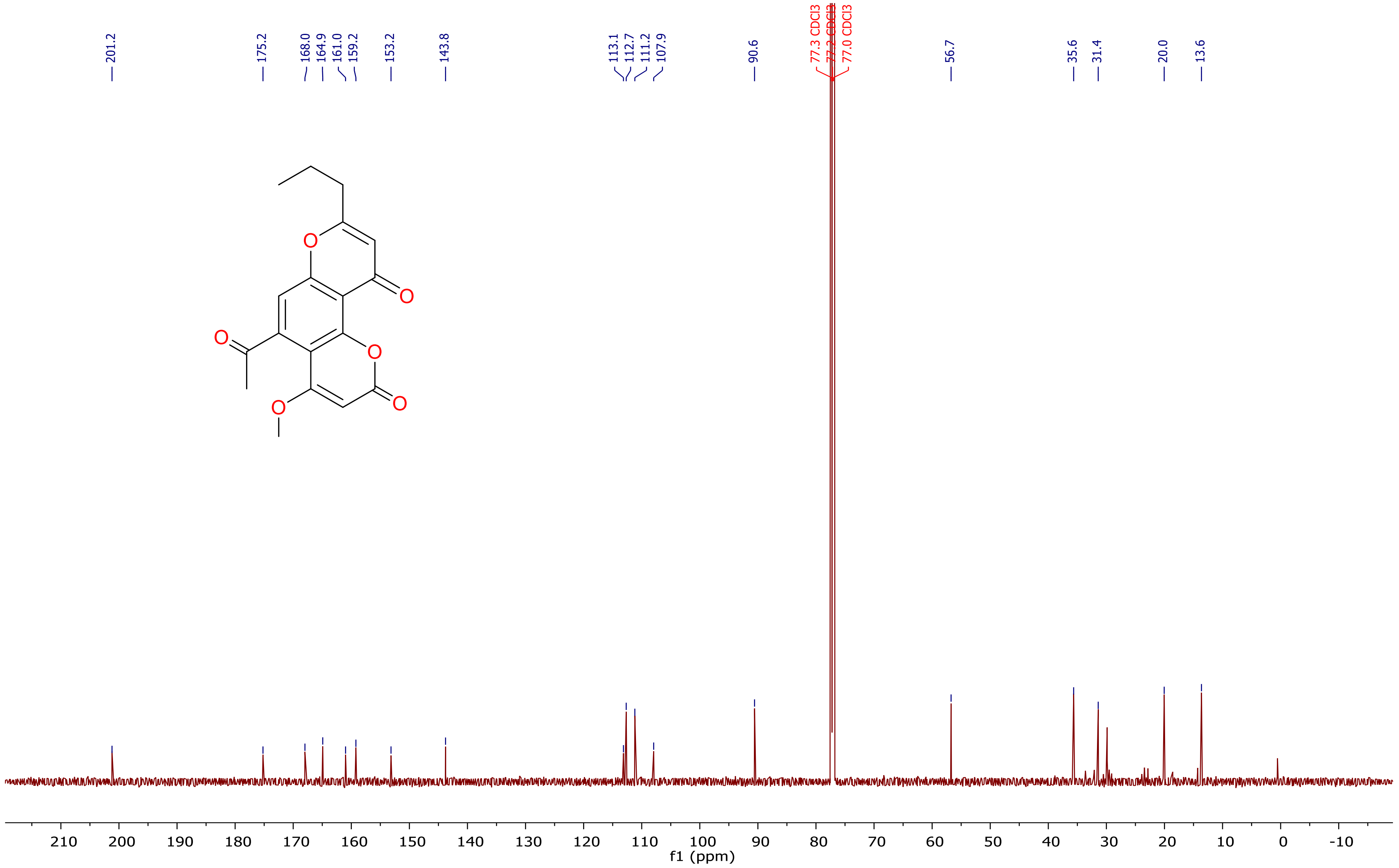

Figure S3 COSY NMR spectrum of capillasterin A (**1**) in $CDCl_3$

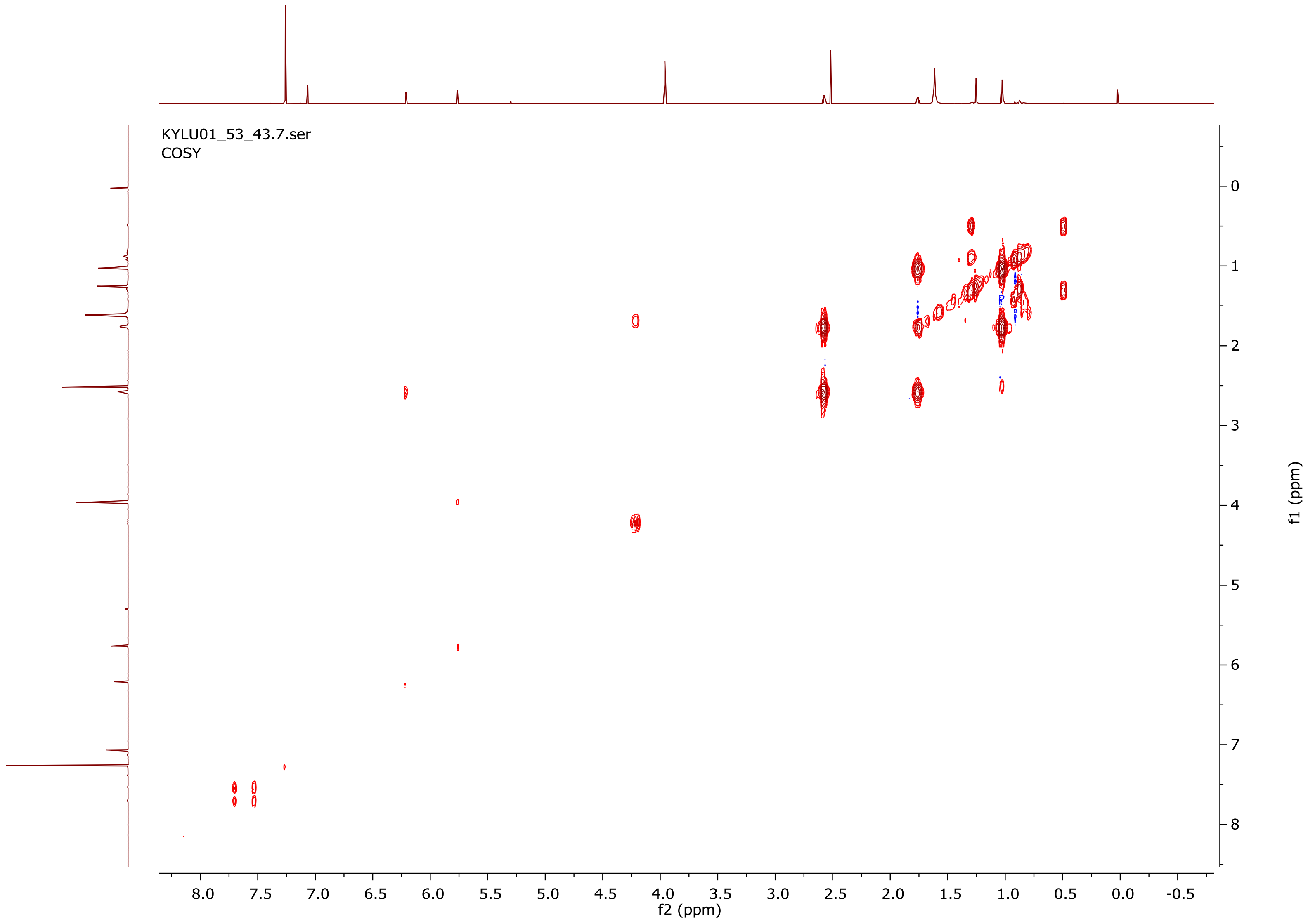

Figure S4 HSQC NMR spectrum of capillasterin A (**1**) in $CDCl_3$

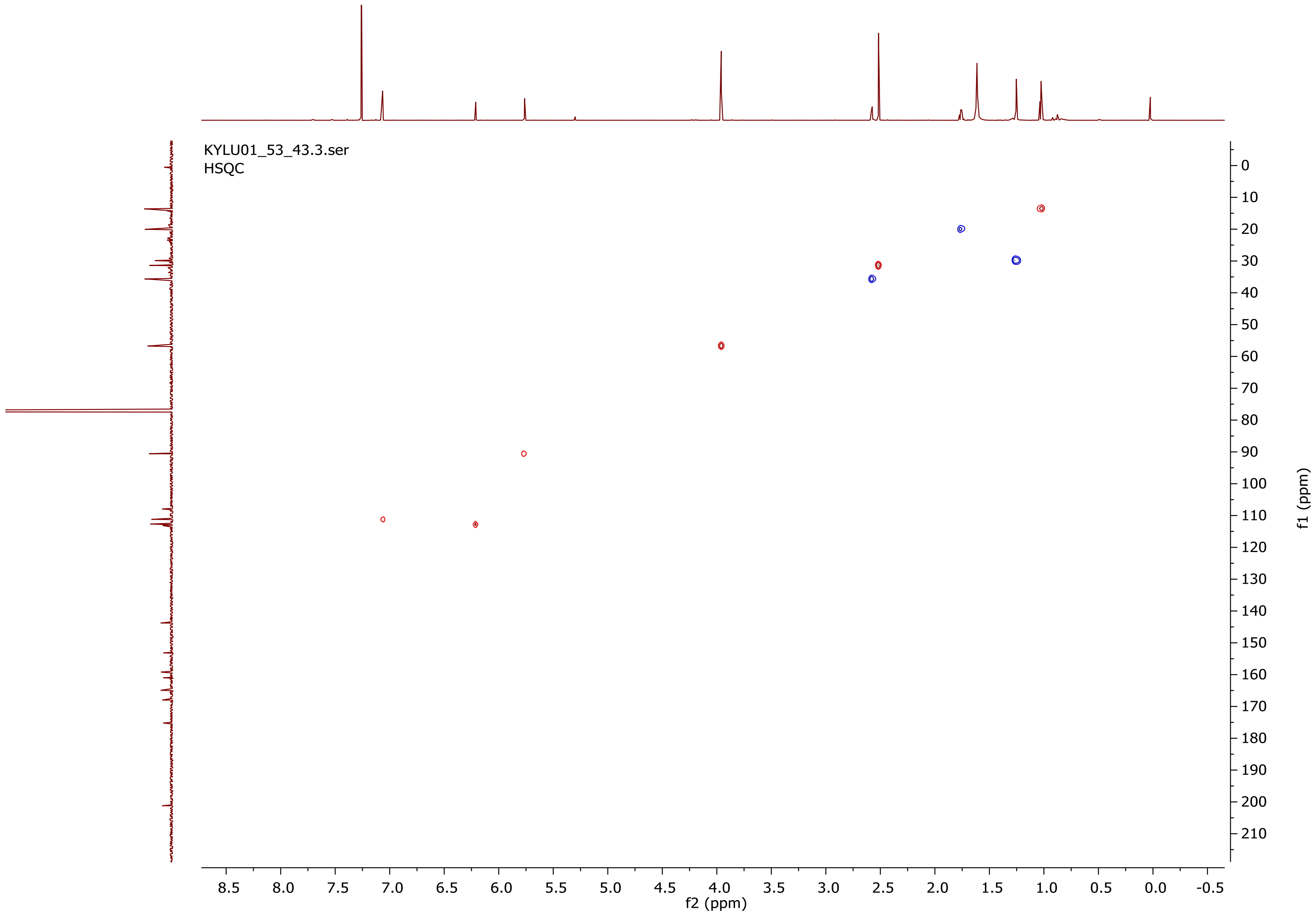

Figure S5 HMBC NMR spectrum of capillasterin A (**1**) in $CDCl_3$

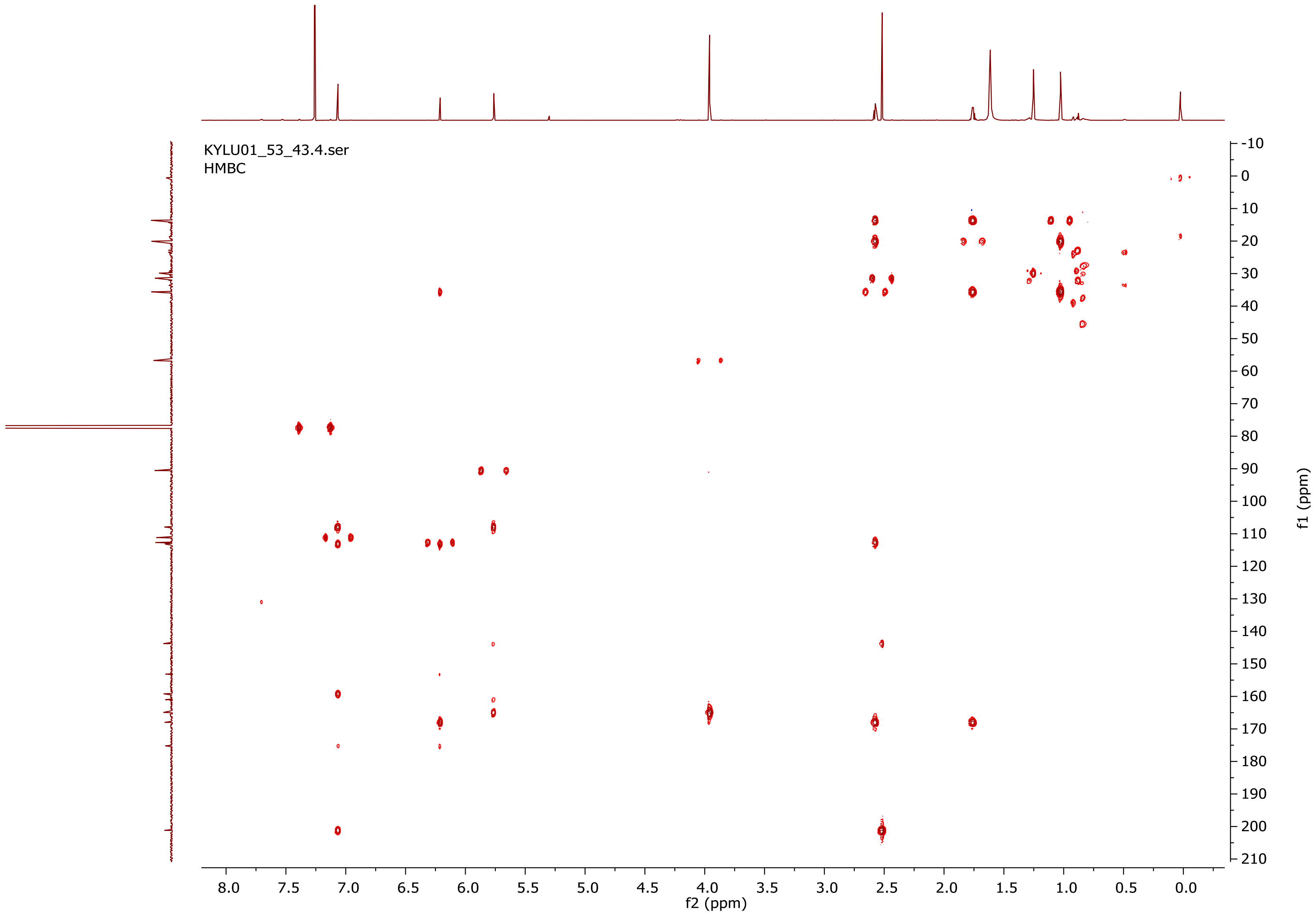

Figure S6 ROESY NMR spectrum of capillasterin A (**1**) in $CDCl_3$

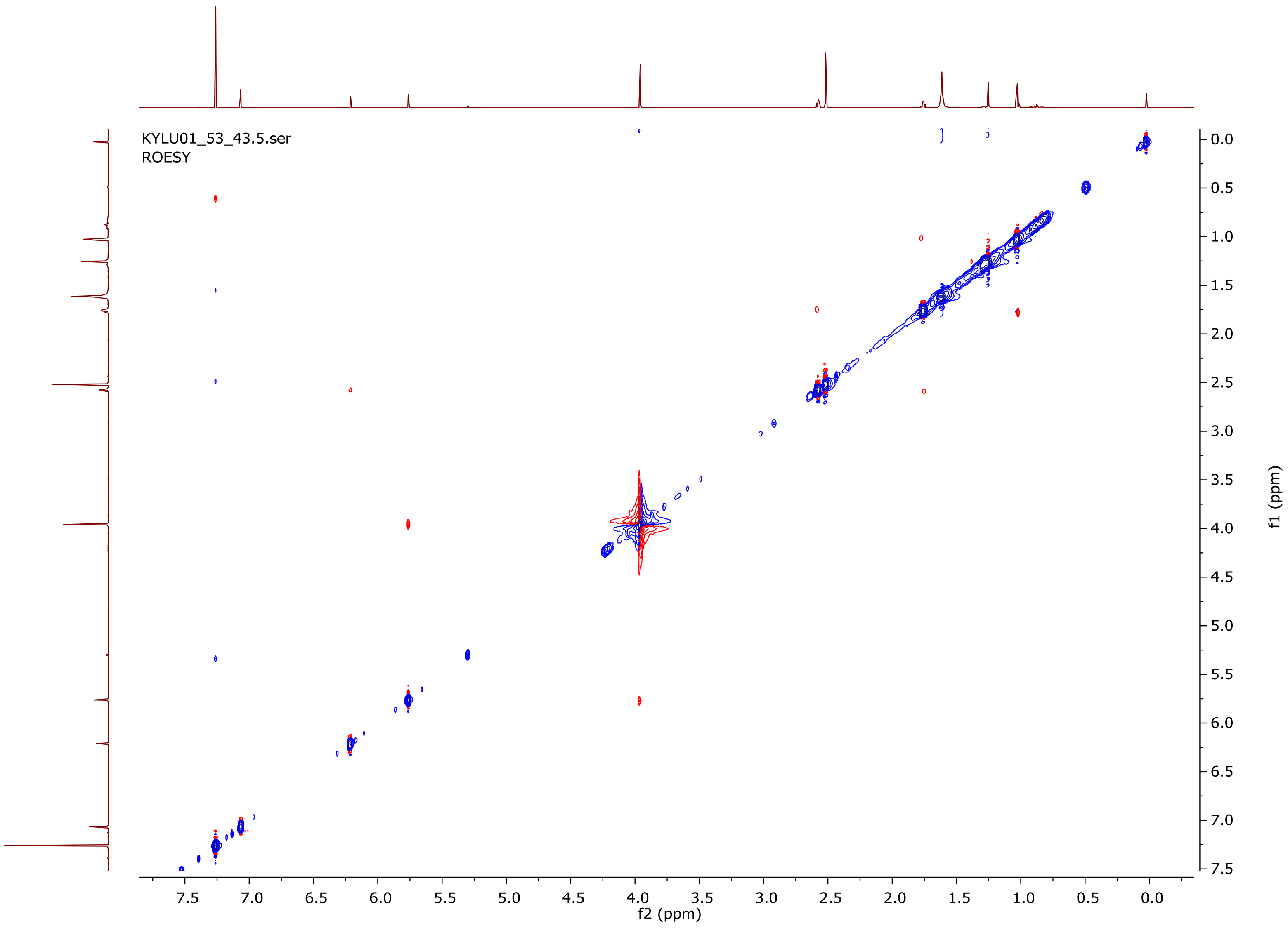

Supplement: Supplementary file 1 [file marinedrugs-17-00026-s001.pdf]
